# Stroke–heart syndrome: current progress and future outlook

**DOI:** 10.1007/s00415-024-12480-4

**Published:** 2024-06-13

**Authors:** Lanjing Wang, Linqing Ma, Changhong Ren, Wenbo Zhao, Xunming Ji, Zhi Liu, Sijie Li

**Affiliations:** 1 Department of Neurology, The People’s Hospital of Suzhou New District, Suzhou, 215129 China; 2 Department of Neurology, Xuanwu Hospital, Capital Medical University, No. 45, Changchun Street, Xicheng District, Beijing, 100053 China; 3 Beijing Key Laboratory of Hypoxic Conditioning Translational Medicine, Xuanwu Hospital, Capital Medical University, Beijing, 100053 China; 4 Clinical Center for Combined Heart and Brain Disease, Capital Medical University, Beijing, 100069 China; 5 Beijing Institute of Brain Disorders, Collaborative Innovation Center for Brain Disorders, Capital Medical University, Beijing, 100069 China; 6 Department of Emergency, Xuanwu Hospital, Capital Medical University, No. 45, Changchun Street, Xicheng District, Beijing, 100053 China

**Keywords:** Remote ischemic conditioning, Stroke–heart syndrome, Acute ischemic stroke, Central autonomic network, Cardiac dysfunction

## Abstract

Stroke can lead to cardiac complications such as arrhythmia, myocardial injury, and cardiac dysfunction, collectively termed stroke–heart syndrome (SHS). These cardiac alterations typically peak within 72 h of stroke onset and can have long-term effects on cardiac function. Post-stroke cardiac complications seriously affect prognosis and are the second most frequent cause of death in patients with stroke. Although traditional vascular risk factors contribute to SHS, other potential mechanisms indirectly induced by stroke have also been recognized. Accumulating clinical and experimental evidence has emphasized the role of central autonomic network disorders and inflammation as key pathophysiological mechanisms of SHS. Therefore, an assessment of post-stroke cardiac dysautonomia is necessary. Currently, the development of treatment strategies for SHS is a vital but challenging task. Identifying potential key mediators and signaling pathways of SHS is essential for developing therapeutic targets. Therapies targeting pathophysiological mechanisms may be promising. Remote ischemic conditioning exerts protective effects through humoral, nerve, and immune-inflammatory regulatory mechanisms, potentially preventing the development of SHS. In the future, well-designed trials are required to verify its clinical efficacy. This comprehensive review provides valuable insights for future research.

**Supplementary Information:**

The online version contains supplementary material available at 10.1007/s00415-024-12480-4.

## Introduction

A systematic global analysis reveals a rising incidence of stroke, and patients with stroke face a substantial risk of developing cardiac complications after onset [1–3]. Acute ischemic stroke (AIS) is the most common type of stroke that deserves special attention. In 1947, Byer et al. first reported that cerebrovascular disease could lead to arrhythmias and myocardial damage [4]. Subsequently, the interaction between brain injury and cardiac function has gained attention, with increasing clinical and experimental evidence supporting a causal relationship between stroke and cardiac dysfunction [5]. Cardiac complications after AIS can result not only from traditional vascular risk factors but also from mechanisms that remain poorly understood. The concept of stroke–heart syndrome (SHS) was proposed in 2018 by Scheitz et al., encompassing stroke-associated cardiac alterations [6].

SHS is categorized into (1) ischemic and non-ischemic acute myocardial injuries; (2) acute myocardial infarction (AMI) after stroke; (3) left ventricular dysfunction (LVD), heart failure (HF), and post-stroke Takotsubo syndrome (TTS); (4) sudden cerebral-cardiac death after stroke; and (5) electrocardiographic (ECG) changes and arrhythmias, including atrial fibrillation (AF) post-stroke [5]. Despite overwhelming evidence suggesting that SHS confers a poor prognosis, its potential pathophysiological mechanisms and therapeutic targets remain unknown.

Therefore, this review aims to present the latest summary of the epidemiology, clinical manifestations, and potential pathophysiological mechanisms of SHS following AIS.

## Epidemiology of stroke–heart syndrome

Patients with AIS have an increased risk of cardiovascular complications. A trial demonstrated that up to one-fifth of patients with AIS developed major cardiovascular adverse events (MACEs) within 12 weeks of stroke onset [7]. A recent retrospective cohort study on more than 360,000 patients with AIS showed that over 27% of them experienced cardiac complications within 4 weeks after stroke [8]. Cardiac complications after stroke are common even in patients without any known history of heart disease. An analysis of 17 studies with 4869 patients with AIS without cardiac history revealed that the mean average incidence rate of asymptomatic coronary artery disease was 52% [9]. And 9.1% of patients with first-time AIS without known preexisting heart disease experienced MACE within 1 year. Compared with individuals without stroke, this population had a 25-fold increased risk of MACE, especially in the first month [10].

The hospitalization-related mortality rate of patients with AIS complicated by ischemic heart disease is approaching 30% [11]. Complex cardiovascular disease accounts for approximately 20% of post-stroke deaths, making it the second most frequent cause of post-stroke death after neurological death [7, 12]. In addition, it is reported that cardiac mortality is the highest in the first month after stroke, especially in the second week [7]. Approximately 4% of patients with AIS die due to cardiac causes, and up to 19% experience at least one MACE [7].

## Manifestations of stroke–heart syndrome

The clinical manifestations of SHS, including asymptomatic ECG abnormalities, malignant arrhythmias, elevated cardiac biomarkers, LVD, and AMI, can develop continuously or occur simultaneously, coexist, or predispose patients to each other [13] (Table 1).

**Table 1:** Summary of clinical manifestation of stroke–heart syndrome

Clinical manifestations	Incidence	Predictors	Outcomes
ECG changes	QTc prolongation: 20–65% [14–21] Abnormal T-wave morphology: 16–40% [14–21] ST-segment changes: 15–25% [14–21] Cardiac arrhythmias: 16.5–22% [16, 17] Atrial fibrillation: 5–24% [21–24]	Older age, severe stroke, and insular infarction [25, 26]	Increase mortality [18, 27]
Myocardial injury	Elevated hs-cTn: 25–60% [6, 28] Acute myocardial injury: 25.3% [28] Acute myocardial infarction: 1–3% [9, 29–33]	Older age, history of cardiac comorbidities, severe stroke, lower low-density lipoprotein levels, renal insufficiency, ECG abnormalities, and insular infarction [25, 34–37]	Poorer neurological outcomes, increased mortality, and cardio-cerebral vascular events recurrence rate [34, 35, 38–40]
Cardiac dysfunction	Impaired left ventricular function with a low ejection fraction: 10–24% [41–44] Clinical heart failure: 5–18% [41–44] Left ventricular diastolic dysfunction: 23–59% [44, 45]	Older age, serious stroke, history of prior cardiac diseases, increased cTn levels, and right insular infarction [44, 46–51]	Poorer neurological outcomes, increased mortality, and cardio-cerebral vascular events recurrence rate [41, 42, 52–55]
Takotsubo syndrome	0.4–2.7% [56, 57]	Older age, Caucasian, and female [57, 58]	Poorer neurological outcomes increased mortality [57, 58]

### Electrocardiographic changes and cardiac arrhythmias

AIS frequently accompanies ECG changes. In patients without preexisting heart disease or clearly altered ECG, approximately one-third exhibit significantly abnormal ECG changes [14]. The most frequently observed post-AIS ECG abnormalities include QTc prolongation (20–65%), abnormal T-wave morphology (16–40%), and ST-segment changes (15–25%) [14–21]. Patients with severe neurological impairment and insular infarction are particularly prone to prolonged QTc [25]. Significant cardiac arrhythmias (CAs) can occur in up to 22% of patients with AIS, with right hemispheric infarcts (26.8%) associated with more frequent CA compared with left hemispheric infarcts (14.3%) [16]. Togha et al. reported that 16.5% of patients with AIS without prior cardiovascular diseases had CA [17]. ECG changes reach a peak early post-stroke, and CA has the highest probability of occurrence within the first 24 h [26]. Older age and higher National Institutes of Health Stroke Scale (NIHSS) score were reportedly independent predictors of CA within the first 3 days after stroke [26]. However, most ECG abnormalities are transient and disappear within 14 days [16, 59, 60]. Tachycardia-associated arrhythmias, especially AF, are more frequent than bradycardias [15, 26]. The incidence of AF following AIS is approximately 5–24% [21–24]. Consistent with the above-mentioned results, the incidence of AF detected after stroke (AFDAS) was higher in strokes involving the right insula (39%) than in strokes involving the left insula (4%) [61]. Notably, cardiac structure and function changes during AF in patients with stroke [62].

ECG abnormalities after AIS correlate strongly with neurological outcomes. Compared with that in patients with normal QTc, the mortality rate triples in patients with prolonged QTc during the early AIS [18]. AF, atrioventricular block, ST-segment changes, and inverted T-waves increase the mortality risk of patients within the subsequent 3 months [27]. AFDAS exhibits unique mechanisms and risk factors. Enhanced left atrial-pulmonary vein border fibrosis may serve as a structural substrate for AFDAS [63]. Patients with initial AFDAS diagnosis, as opposed to those with prior AF, may have a lower incidence of cardiac comorbidities and recurrent AIS [64, 65]. Nevertheless, Yang et al. reported similar AIS recurrence and mortality rates between these patient groups [66].

### Cardiac troponin elevation

Increased cardiac troponin (cTn) levels are closely associated with acute myocardial injury, which is typically defined as a cTn level increase exceeding the reference superior limit, with a change of > 20% [67, 68]. Elevated cTn levels often occur without typical symptoms, emphasizing the importance of timely measurement upon admission for AIS [69]. The overall incidence of post-AIS myocardial injury is 25–60% with highly sensitive cardiac troponin (hs-cTn) [6, 28]. In addition, 25.3% of patients were diagnosed with acute myocardial injury after stroke [28]. Typically, elevated cTn levels appear transient, returning to normal range within hours to days. cTn assessment is pivotal in AMI diagnosis. The initial cTn level within 4.5-h post-stroke symptom onset demonstrates enhanced diagnostic efficiency for AMI, with 90.9% sensitivity and 74.8% specificity [70]. Previous studies reported that 2.3% of AIS patients were diagnosed with AMI during hospitalization, 1.6% of AIS patients were diagnosed with non-ST-elevation AMI, and 0.3% were diagnosed with ST-elevation AMI [29–31]. AMI occurs in 1–3% of patients with AIS within 1 year, with the highest risk in the first month and gradually decreasing thereafter [9, 32]. The cumulative 5-year incidence of post-stroke AMI is 2% in Korea [33]. Increased cTn levels may be associated with older age, a history of cardiac comorbidities, severe stroke, lower low-density lipoprotein (LDL) levels, renal insufficiency, and ECG abnormalities [25, 34, 35]. Stroke affecting the right insula is highly correlated with increased cTn levels [36, 37]. Extremely high cTn levels are also associated with heart dysfunction, decreased left ventricular ejection fraction (LVEF), and segmental ventricular hypokinesia [61]. Blaszczyk et al. observed that over 30% of patients with AIS developed focal myocardial fibrosis, primarily with an acute or subacute ischemic pattern, using cardiovascular magnetic resonance (CMR) imaging [71].

Animal experiments have demonstrated a fourfold increase in cTn level in mice 24 h after middle cerebral artery occlusion (MCAO), correlating with elevated mortality [72]. High initial and peak cTn levels are associated with poorer neurological outcomes and increased mortality, with dynamic cTn changes doubling in-hospital death risk [35]. Patients with AIS who have high cTn levels have a mortality rate of 14.7%, which is six times higher than that in patients with normal levels [34]. Elevated hs-cTn levels increased the incidence of death and major disability within 90 days [38]. Moreover, elevated cTn levels impact long-term prognosis, potentially increasing mortality related to stroke, heart disease, and cancer [39]. A recent study suggests that elevated hs-cTn levels are a valuable biomarker for cardio-cerebral vascular events recurrence and mortality [40].

### Cardiac dysfunction

Numerous studies indicate that AIS can cause cardiac dysfunction. Min et al. induced left insular cortex ischemia in mice, resulting in cardiac dysfunction [73]. Veltkamp et al. observed a transient decrease in left ventricular contractility in early AIS stages in mice, recovering after 2 months [74]. Nevertheless, chronic systolic dysfunction was detected 8 weeks after focal cerebral ischemia in mice [75]. Clinical research suggests that approximately 10–24% of patients experience impaired left ventricular function with a low ejection fraction (EF) [41–44]. Approximately 13–15% of patients have moderate/severe left ventricular systolic dysfunction (EF < 40%) [43, 76]. Clinical HF is diagnosed in approximately 5–18% of patients with AIS, mostly with preserved EF [41–44]. Left ventricular diastolic dysfunction reportedly affects 23–59% of patients with AIS [44, 45]. Older age, serious stroke, history of prior cardiac diseases, and increased cTn levels are predictive factors for LVD. [44, 46–48] The size and area of stroke strongly correlate with the severity of cardiac dysfunction [49]. LVD is closely linked to damage in the right hemispheric central autonomic network (CAN) [50]. Infarcts in the right insula and left parietal cortex are more likely to result in LVD in patients with AIS without preexisting cardiac dysfunction [51].

The hospitalization mortality rate is 2.5 times higher in stroke patients with HF than in non-HF patients [52]. Regardless of EF reduction, HF correlates with poor neurological outcomes and 90-day mortality after stroke [41]. Lower LVEF independently predicts 90-day disability and increases the incidence of MACE and 1-year all-cause mortality post-stroke onset [42, 53]. However, abnormal echocardiographic results were not related to short-term mortality but significantly correlated with mortality 3 years after stroke [54].

Brain natriuretic peptide (BNP) and N-terminal pro-B-type natriuretic peptide (NT-proBNP) serve as predictors of post-stroke cardiac dysfunction [77]. NT-proBNP levels significantly increased within 24 h of AIS onset, whereas BNP correlated with left ventricular hypertrophy, left atrial dilatation, and LVEF [78, 79]. NT-proBNP levels are independently associated with all-cause mortality at 90 days [55].

TTS is a reversible form of HF, previously termed “stress-induced cardiomyopathy”, typically resolving within 1–6 months [5]. Post-stroke TTS involves transient ventricular dysfunction, with or without increased cTn and ECG changes [56]. Acute hemodynamic changes in TTS include depressed cardiac contractility, abbreviated systolic period, inefficient energetics, and extended active relaxation but unaltered diastolic passive stiffness [80]. TTS following AIS occurs in 0.4–2.7% of patients, with older, Caucasian, and female patients at increased risk [56–58]. Reduced LVEF and elevated neutrophil-to-lymphocyte ratio increase in-hospital complications and reduce long-term survival in TTS patients [81, 82]. TTS secondary to AIS predicts poorer short-term neurological functional outcomes and high mortality, with twice the inpatient mortality compared to patients without TTS [57, 58].

## Pathophysiology of stroke–heart syndrome

SHS is caused by various pathophysiological mechanisms inductively defined as “stroke-induced heart injury (SIHI)” [5]. Evidence provides compelling support for CAN dysregulation, hypothalamic–pituitary–adrenal (HPA) axis activation, and inflammation in SHS [5, 6].

**Fig. 1:**
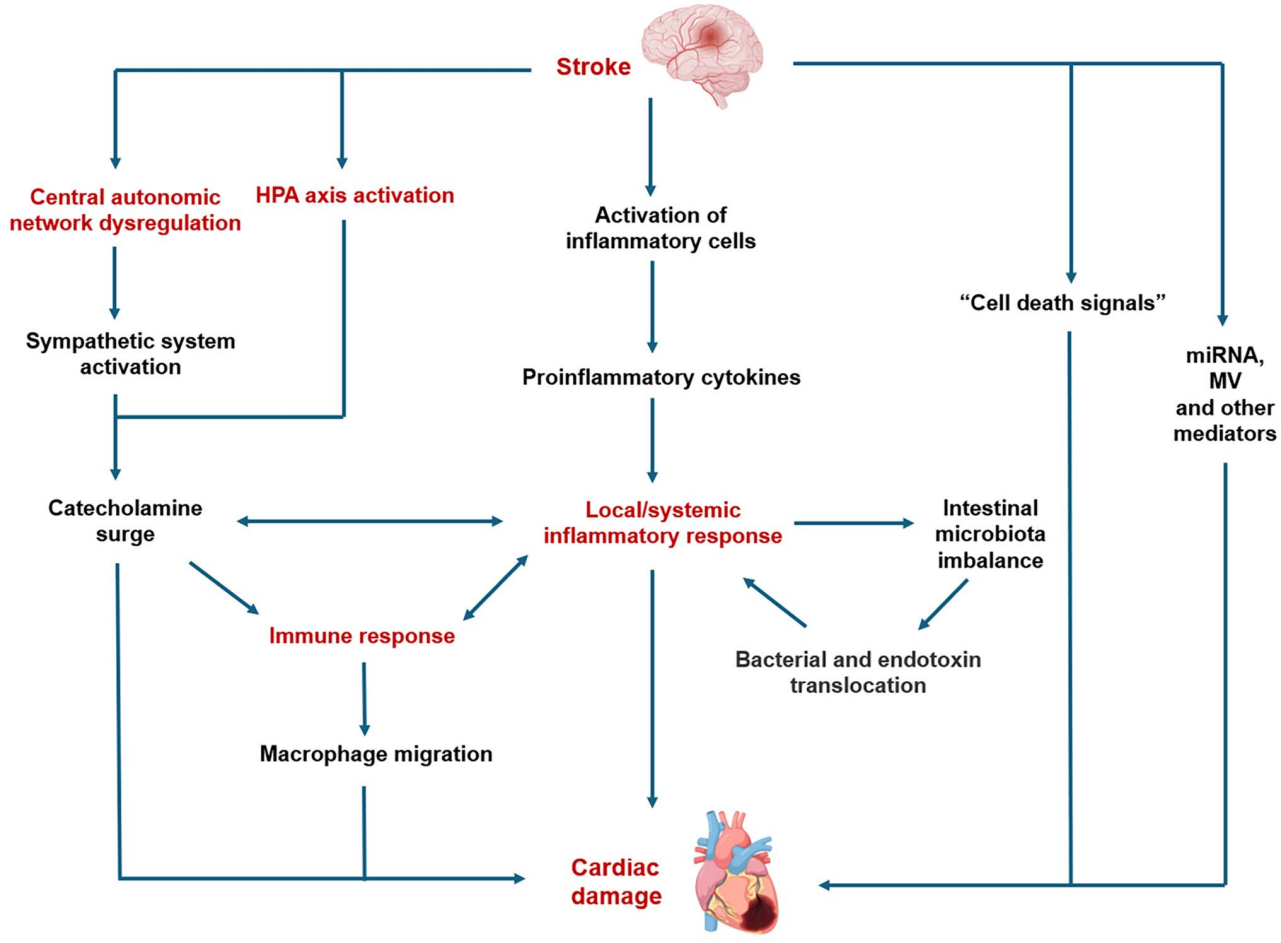
Mechanisms for stroke–heart syndrome. Stroke leads to central autonomic network dysregulation, HPA axis activation, inflammation, and immune response. These mechanisms interact with each other, ultimately resulting in cardiac damage. “Cell death signals”, miRNA, and MV are also involved in the development of SHS. *HPA* hypothalamic–pituitary–adrenal, *MV* microvesicle

AIS can lead to damage to the CAN, resulting in overactivity of the sympathetic nervous system and HPA axis, further causing a surge in catecholamines [5, 6]. AIS leads to neuronal cell necrosis, activation of inflammatory cells, and the release of inflammatory response mediators, leading to local and systemic inflammation [83]. The peripheral immune system initiates after AIS, leading to macrophage migration [84]. There can be interactions between the autonomic nervous system, inflammatory response, and immune response [85]. Macrophages secrete cytokines and chemokines to promote the migration of inflammatory cells [84]. The surge in catecholamines enhances immune regulation and mediates an increase in inflammatory cytokines, aggravating the inflammatory response [85]. Simultaneously, inflammation also affects the release of catecholamines from the posterior hypothalamus and sympathetic nerve [85]. The increase in cortisol levels and inflammatory response caused by post-stroke stress response can increase intestinal barrier permeability, allowing the translocation of bacteria and endotoxins into the blood, further inducing an inflammatory response and the production of pro-inflammatory cytokines [84]. The interactions between these mechanisms jointly lead to cardiac damage (Fig. 1).

### Central autonomic network dysregulation and hypothalamic–pituitary–adrenal axis activation

The CAN involves various brain structures, including the insular cortex, anterior cingulate cortex, prelimbic and infralimbic areas, cingulate cortex, amygdala, and bed nucleus of the stria terminalis [86]. Damage to these sites disrupts the CAN structure and function and can result in a hyperactive stress response involving the sympathetic nervous system and HPA axis [5, 6]. Min et al. found that 64% of mice developed cardiac dysfunction after left MCAO, whereas those undergoing right MCAO did not [73]. Moreover, the severity of left insular cortex infarction in mice with impaired cardiac function was obviously greater [73]. Bieber et al. showed that mice undergoing right hemispheric MCAO developed mild cardiac dysfunction, whereas left MCAO-treated mice did not show such changes [75]. The mice with cardiac dysfunction had more severe insular cortex damage and increased sympathetic activity [75]. Moreover, larger insular infarct volumes correlate with more severe myocardial injury [87]. Meanwhile, this evidence supports the notion that the lateralization of the insula is closely connected with autonomic responses. Research suggests that the right insular cortex focuses on the sympathetic nervous system, whereas the left insular cortex is involved in parasympathetic function [88–90]. Heart rate variability (HRV) analysis studies reveal that patients with left hemisphere damage show enhanced parasympathetic regulation, whereas those with right hemisphere ischemia show the opposite effect [89, 90].

Following overactivation of the sympathetic nervous system and HPA axis, catecholamines and cortisol are excessively released, impacting the heart [6]. The expression of norepinephrine in the serum and heart tissue significantly increases, with cardiac contraction band necrosis observed in mice experiencing cardiac dysfunction during the acute stage of stroke [73]. Cardiac dysfunction is not exclusive to the acute stage of AIS. Accumulating evidence indicates that cerebral ischemia can lead to chronic cardiac dysfunction [75, 91]. In an animal experiment, compared to 3 days after stroke, 1 month showed more significant and severe cardiac dysfunction and myocardial pathological remodeling [74]. Chronic systolic dysfunction was observed in both young and elderly mice at 8 weeks after MCAO. Elevated catecholamine and cortisol levels suggest the involvement of enhanced sympathetic activity and indicate that the influence of AIS on cardiac function is age-independent [75]. Hypertension may modify central control of the CAN, as seen in spontaneously hypertensive rats exhibiting significant decreases in arterial pressure and renal sympathetic nerve discharge 6 h after MCAO [92]. Excessive calcium influx into cardiomyocytes during catecholamine storms can induce cardiac electrical instability and CA [6]. Sun et al. noted higher L-type calcium current density in ventricular myocytes of MCAO rats with arrhythmias, attributed to upregulated mRNA and protein expression of α_1C_/Ca_v_1.2, enhancing the L-type Ca^2+^ channel function [93]. Another study reported that significant CA primarily results from increased L-type Ca^2+^ current and reduced transient outward K^+^ current [94].

### Immune response and inflammation

Inflammatory responses and immune regulation significantly impact stroke–cardiac interactions [72, 84, 85, 95]. Ischemic stroke (IS) induces systemic and local cardiac inflammation and causes acute HF with increased circulating cTn levels and bradycardia [72, 96, 97]. Interleukin (IL)-1 is pivotal in SIHI by enhancing the inflammatory response [5]. Cerebral ischemia can directly damage neurons, activate microglia, and release inflammatory factors such as IL-1, stimulating cardiac macrophage and myofibroblast [5, 98]. Splenectomy before cerebral infarction in mice alleviates early inflammatory responses and heart infiltration, suggesting the peripheral immune regulation of the spleen after stroke plays an important role in post-stroke cardiac dysfunction [84]. Persistent macrophage infiltration from acute to chronic stroke phase indicates its crucial role in brain–cardiac interactions [84]. Inflammatory mechanisms in chronic stroke may be attributed to the weakened protective benefit of the inflammatory reflex against the post-autonomic dysfunctional inflammatory response [63, 99].

Although inflammatory factors emerge rapidly after cerebral ischemia, their circulation is temporary, whereas myocardial remodeling can persist for weeks or months [5]. Systemic inflammation and sympathetic hyperactivity activate microvascular endothelial cells, causing coronary microvascular endothelial dysfunction (CMED) [100]. An experimental stroke model demonstrated the combined role of intercellular communication and pro-inflammatory signaling in brain–heart interactions [91]. Balint et al. detected increased endothelial nitric oxide synthase (eNOS)-expressing endothelial cells in the left atrium of mice 28 days after stroke, indicating CMED [63]. Insular cortical infarction causes myocardial inflammatory infiltration and fibrosis in the left atrial, with the severity of CMED and cardiac fibrosis being associated with the level of inflammation [63].

### Potential molecular mechanism and signaling pathways of the stroke–heart syndrome

MicroRNA (miRNA)-126 regulates endothelial cell (EC) function, vascular remodeling, vascular integrity, and endovascular inflammation [101]. Deletion of miRNA-126 in mice resulted in impaired cardiac function, cardiomyocyte hypertrophy, fibrosis, inflammatory reactions, and oxidative stress after stroke [91]. Activation of the NLR pyrin domain-containing 3 (NLRP3) inflammasome also mediates brain–heart interactions post-stroke [102]. Elevated NLRP3 expression in diabetic stroke mice correlated with impaired cardiac function partially reversible by NLRP3 inhibitors [102]. Veltkamp et al. showed that left MCAO in mice exhibited rapid cardiomyocyte atrophy via upregulated E3-ligase atrogin-1 and proliferator-activated receptor gamma (Pparg)-dependent genes. Pparg regulates cardiomyocyte metabolic and structural remodeling, supporting the latent role of metabolic signaling pathways in SHS [74]. Notably, stroke diminishes cardiac function and increases cardiac vulnerability to ischemia [103]. Cerebral ischemia altered nitro-oxidative signaling in the heart, affecting the expression of eNOS and glutathione peroxidase 1 and modulating the survivor-activating factor enhancement cardioprotective signaling pathway [103].

Indirect “cell death signals” from the brain to the heart were confirmed in vitro and in vivo [104]. Supernatants from rat neuronal cells exposed to oxygen–glucose deprivation increased cell death markers in rat cardiac myocytes (RCMs), reducing their vitality and mitochondrial reductase activity. An in vivo study revealed RCM cell death in rats 3 months after MCAO and abnormal inflammatory responses and apoptosis in non-human primates 6 months after transient global ischemia [104, 105].

## Future directions

### Early prediction and identification of SHS

Approximately one-fifth of patients with AIS show persistent SHS symptoms [6, 106]. Prevention and early identification is crucial. Tumor necrosis factor-related apoptosis-inducing ligand expression correlates with premature ventricular extrasystoles after stroke [107]. Soluble ST2 predicts SHS severity, and a risk prediction scale incorporating various factors identifies high-risk patients with SHS [108].

Continuous cardiac monitoring is crucial for detecting serious CA and preventing sudden cardiac death. A retrospective study reported the effectiveness of continuous monitoring in detecting underlying clinical AF during the 6-month follow-up [109]. Dynamic analysis of the RR interval performed in the hyperacute phase of AIS can help identify high-risk paroxysmal AF episodes [110]. Several predictive models have been developed for AFDAS [23, 111]. Further, a study established a multimodal approach incorporating imaging, ECG, and original biomarkers [112].

In addition, CMR imaging may offer superior accuracy in detecting subtle cardiac changes related to cardiac ischemic processes [113]. The PRAISE (PRediction of acute coronary syndrome in Acute Ischemic Stroke) study is refining algorithms and developing guidelines based on CMR findings to improve the identification of AIS patients at risk of SHS [114].

### Autonomic function assessment

Autonomic dysfunction, persisting for 6 months after stroke, plays a crucial role in SHS development [115]. Therefore, it is necessary to assess post-stroke cardiac dysautonomia. HRV analysis shows promise in predicting mortality and morbidity in stroke outcomes [90, 116–119]. Phase-rectified signal averaging is also an ECG-based method for evaluating cardiac autonomic function, which reflects sympathetic and vagal nerve activities by calculating the acceleration (AC) and deceleration (DC) capabilities of the heart rate [120]. Unfortunately, we are still far from realizing these approaches are very important unmet needs [121]. CORONA-IS (Cardiomyocyte injury following Acute Ischemic Stroke) study is addressing quantifying autonomic dysfunction [122].

### Exploring underlying mechanisms and mediators

Brain-derived neurotrophic factor (BDNF) may impact SHS [123]. Circulating BDNF levels are decreased after stroke [124]. BDNF can bind to tropomyosin-related kinase receptor B and trigger a calmodulin-dependent protein kinase II-dependent signaling cascade, which enhances cardiomyocyte Ca^2+^ cycling [123]. MiRNA regulates various biological processes, including hypoxia responses, angiogenesis, and inflammation [13]. MiRNA-182 dysregulation is linked to multiple cardio-cerebrovascular diseases, with elevated levels in AIS patients and cardiovascular disease patients [125]. A relationship between BDNF and miRNA-182-5p has been reported in HF patients [126]. MiRNA-124-3p, a brain-enriched miRNA, is upregulated after cardiac arrest, correlating with neurological outcomes [127]. Circulating microvesicles (MVs) may also influence brain–heart interactions [13]. MVs are considered a potential biomarker for cardiovascular risk stratification [128]. In the initial stage of AIS, the endothelial MVs and leukocyte-derived MVs levels are elevated, and endothelial MVs were proved to be associated with stroke severity [129]. A prospective cohort found that elevated endothelial MV and leukocyte-derived MV levels were interrelated to the long-term risk of cardiovascular events in stroke patients [130]. Therefore, future studies are warranted on the effects of miRNAs and MVs in the development of SHS, which deserve further study.

Intestinal microbiota imbalance is implicated in SHS development [13, 83]. AIS disrupts intestinal integrity, alters microbiota composition, disrupts immune homeostasis, and leads to the loss of intestinal neurons [131]. Increased intestinal permeability may trigger inflammatory reactions, exacerbating cardiac function [84]. Dysbiosis and gut microbiota translocation may promote inflammation post-myocardial ischemia/reperfusion, worsening heart damage [132]. Nevertheless, the detailed mechanism of gut microbiota in SHS remains unclear, necessitating future investigation.

### Development of treatment strategies

AMI after AIS presents a crisis situation. Early-stage treatment of AIS and AMI primarily relies on intravenous thrombolysis therapy or endovascular therapy to achieve recanalization and salvage ischemic tissue, but the thrombolytic drug doses and treatment time window differ between these two cases [133]. For synchronous cardio-cerebral infarction, clear guidelines or robust evidence for formulating acute reperfusion strategies are lacking. According to the 2019 recommendations from the American Heart Association/American Stroke Association, administering intravenous thrombolysis with alteplase (AIS therapeutic dose) within 4.5 h, followed by percutaneous coronary intervention (if necessary), may be reasonable [134]. However, the recommendation lacks specific, and it is safer and more effective to provide individualized treatment schemes after evaluating the severity of cardio-cerebral events in patients with cardio-cerebral infarction.

**Fig. 2:**
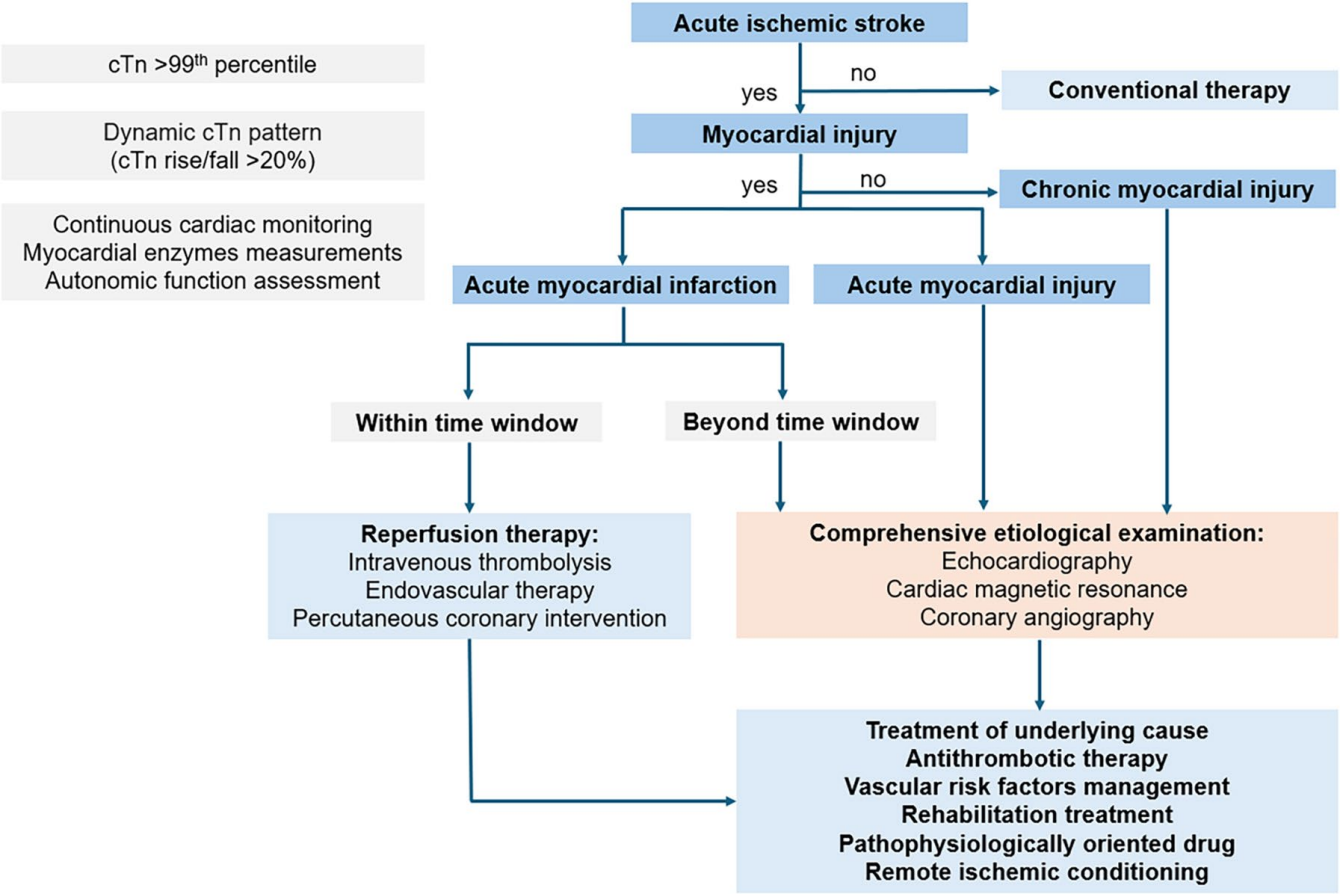
Diagnosis and management of myocardial injury after acute ischemic stroke. Continuous cardiac monitoring, myocardial enzyme measurements, and autonomic function assessment are necessary after stroke onset. If no contraindications exist, patients with acute myocardial infarction after acute ischemic stroke (within the time window) can be treated with reperfusion therapy. Patients with other types of myocardial injury should undergo a comprehensive etiological examination followed by treatment of the underlying cause

At the same time, developing effective treatment strategies for SHS and its long-term consequences is vital [135]. Recently, researchers proposed a thorough and comprehensive care approach following the post-stroke ABC pathway to prevent, identify, and treat SHS [136]. The ABC pathway includes three prominent aspects: (A) appropriate antithrombotic therapy; (B) improvement in functional and psychological status; and (C) management of cardiovascular risk factors and comorbidities [137]. Stroke and cardiac diseases share various risk factors and pathophysiological mechanisms [138]. Although antithrombotic therapy is essential for both conditions, its benefits in preventing recurrent vascular events remain unclear [139]. A cohort study demonstrated similar long-term results of antithrombotic tactics in patients with AIS and HF (EF ≤ 40%) [140]. Adequate management of vascular risk factors is vital for preventing recurrent vascular events. Recent guidelines endorse targets for blood pressure, LDL cholesterol and glycated hemoglobin levels in patients with AIS, but optimal criteria for those with different cardiac complications remain unknown [141]. In addition, cardiac manifestations caused by non-atherosclerotic factors require a comprehensive examination to identify the etiology and subsequently treat the underlying causes [5] (Fig. 2).

SHS involves unique mechanisms beyond traditional risk factors, suggesting the benefits of therapies targeting sympathetic nervous system modulation, inflammation, vascular endothelial function improvement, and avoiding proarrhythmic drugs [100]. However, data on β-blockers, IL-1 receptor antagonists, and renin–angiotensin system inhibitors are insufficient for strong recommendations [5, 100]. Remote ischemic conditioning (RIC) has emerged as a safe and promising physical therapy for cardio-cerebrovascular diseases, with reported protective effects through humoral, nerve, and immune-inflammatory regulation mechanisms [142–148]. A recent study confirmed that RIC reduced the recurrence rate of vascular events and improved 90-day neurological outcomes in patients with AIS and AMI [149]. Therefore, early application of RIC post stroke onset may help prevent the development of SHS.

In conclusion, interdisciplinary cooperation is essential for the development and implementation of SHS treatment strategies. Future studies should focus on identifying key mediators and signaling pathways of brain–heart interaction to pinpoint therapeutic targets and improve patient outcomes. Recognizing SHS more deeply and conducting relevant clinical and animal studies are encouraging steps toward addressing this complex condition.

### Supplementary Information

Below is the link to the electronic supplementary material.

Supplementary file1 (DOCX 54 kb)
