# Supplementary material for: Stroke–heart syndrome: current progress and future outlook
Source: J Neurol. 2024 Jun 13;271(8):4813–25. doi: 10.1007/s00415-024-12480-4 (PMC11319391; doi:10.1007/s00415-024-12480-4)
Supplement: Supplementary file 1 — Supplementary file1 (DOCX 54 kb) [file 415_2024_12480_MOESM1_ESM.docx]

**sTable 1** Summary of animal experiments on the mechanism of stroke–heart syndrome.

| **Animal** | **Model** | **Observation Indicators** | **Results** | **Mechanisms** | **Ref.** |
| --- | --- | --- | --- | --- | --- |
| Male C57BL/6 mice (12 weeks) | Ischemia model via left/right permanent MCAO | *24 hours after pMCAO:*  cardiac function,  infarct volume,  serum and heart levels NE. | The severity of left insular cortex lesion was greater in mice with cardiac dysfunction. Serum and heart NE elevated in mice with cardiac dysfunction. | Excess NE release may mediate this cardiac dysfunction. | ^1^ |
| Male C57BL/6 mice (8–12 weeks) | Ischemia model via 30 min/60 min left MCAO | *1~14 days after MCAO:*  cardiac function,  serum hsTNT,  transcript levels of nppa, nppb, col5a1, atrogin-1, murf-1 in the hearts,  heart levels NE; | Left ventricular contractility was reduced early but not late after brain ischemia. Net norepinephrine was increased. The E3‐ligase atrogin‐1 and Pparg‐dependent genes up‐regulated. | Brain ischemia triggers a disturbance of catecholamine homeostasis in the heart, leading to immediate but transient cardiac atrophy and dysfunction. Pparg is a potential mediator of stroke‐induced transcriptional dysregulation involved in cardiac atrophy. | ^2^ |
| Male C57BL/6J mice  (6‐8 weeks or 1 year) | Ischemia model via 30-min left/right transient MCAO | *1, 3, 8 weeks after MCAO:*  cardiac function;  *8 weeks after MCAO:*  plasma BNP, epinephrine, NE, and cortisol Levels. | tMCAO‐treated mice presented significantly increased levels of plasma norepinephrine, epinephrine, and cortisol. Metoprolol reduced catecholamine levels of norepinephrine and epinephrine and cortisol levels in the blood plasma compared with vehicle‐treated mice. | Focal cerebral ischemia in mice leads to the development of chronic systolic dysfunction driven by increased sympathetic activity. Metoprolol treatment prevented the development of chronic cardiac dysfunction. | ^3^ |
| Male C57/BJ6L mice (3–4 months) | Ischemia model via right permanent dMCAO | Generation of specific conditional EC miR-126 knockout (MiR-126 ^EC−/−^) mice;  *4 weeks before and after MCAO:*  cardiac function;  *4 weeks after MCAO:*  myocyte cross-sectional area,  interstitial collagen fraction,  positive areas of PSR, TGF-β, NOX2, MCP-1 and positive cell number of ED1, concentration of VCAM-1, PSR, TGF-β, NOX2, MCP-1,  MiR-126 expression. | Stroke mice exhibited significantly decreased cardiac ejection fraction and increased myocyte hypertrophy, fibrosis as well as increased heart inflammation, infiltrating macrophages, and oxidative stress.  MiR-126^EC−/−^ mice exhibited significantly decreased cardiac function and increased cardiomyocyte hypertrophy, fibrosis, and inflammatory factor expression after stroke compared to miR-126 ^fl/fl^ stroke mice. | Decreasing miR-126 expression may contribute to cardiac dysfunction after stroke in mice. | ^4^ |
| SHR rats and Wistar rats (18–23 weeks) | Ischemia model via right MCAO | *20 min before MCAO and at 1, 3 and 6 h after MCAO:*  catecholamine assay;  *6 hours after MCAO:*  infarct volume. | The central control of the autonomic nervous system is different between SHR and normotensive rats.  Ischemic damage results in a fall in renal sympathetic nerve discharge, adrenal activity, and arterial pressure in SHR. | These differences in the autonomic reaction to MCAO may reflect genetic differences in the response to cerebral ischemia. | ^5^ |
| Wistar male rats | Ischemia model via right/left MCAO | *2 hours after MCAO:*  ECG,  L-type Ca^2+^ current,  the mRNA and protein expression of α_1C_/Ca_v_1.2. | The normalized peak currents of I_Ca,L_ in ventricular myocytes were larger in cerebral ischemic rats with arrhythmias than those in sham-operated rats. The mRNA and protein expression of α_1C_/Ca_v_1.2 was also increased in the myocardium of cerebral ischemic rats with arrhythmias. | The up-regulation in function and expression of L-type Ca^2+^ channel as well as intracellular calcium increasing in cardiac myocytes of cerebral ischemic rats may provide mechanisms of arrhythmogenesis. | ^6^ |
| Wistar male rats | Ischemia model via right MCAO | *Continuously throughout the experiment：*  measure QT Interval;  *24 hours after MCAO:*  L-type Ca^2+^ current, K^+^ currents  protein and mRNA levels of Na^+^ ion/K^+^ ion/ Ca^2+^ ion channel subunits. | Peak transient outward K^+^ current at +60 mV was found to decrease by ~ 32.3% (P < 0.01) in cerebral ischemic rats. The peak amplitude of I_Ca,L_ was increased, and the inactivation kinetics were slowed (P < 0.01). The protein level of the pore-forming subunit for Ito was decreased, but that for I_Ca,L_ was increased. | The depression of Ito and the increase of I_Ca,L_ are the major ionic contributors to arrhythmias after cerebral ischemia. | ^7^ |
| Male C57BL/6 J mice  (12–14 weeks) | Ischemia model via right tMCAO | *24 hours after pMCAO:*  cardiac function,  plasma hsTNT, catecholamines, cytokines. | Systolic left ventricular dysfunction occurred with impaired global longitudinal strain, lower blood pressure, reduced stroke volume, and severe bradycardia, leading to reduced cardiac output. This was accompanied by a systemic inflammatory response characterized by granulocytosis, lymphopenia, and increased levels of serum-amyloid P and IL-6. | Ischemic stroke leads to acute heart failure, bradycardia, systemic, and local inflammatory responses. | ^8^ |
| Male C57BL/6 J mice | Photothrombotic stroke  with/without splenectomy | *3 days and 1 month after stroke:*  measure cardiac function,  gene expression of IL-1β, IL-6, MCP-1, ED1;  Positive areas of PSR, MCSA, and the number of positive cells of MCP-1, TGF-β, IBA1, NK and CD3;  *1 month after stroke:*  cardiac fibrosis and cardiomyocyte size. | Stroke significantly increases macrophage infiltration into the heart and increases IL-1β, IL-6, MCP-1, TGF-β, and macrophage-associated inflammatory cytokine levels in the heart, as well as induces cardiac fibrosis and hypertrophy. Splenectomy with stroke significantly reduces macrophage infiltration into the heart, decreases inflammatory factor expression in the heart, decreases cardiac hypertrophy and fibrosis, as well as significantly improves cardiac function compared to non-splenectomized adult stroke mice. | Cerebral ischemic stroke in adult mice induces chronic cardiac dysfunction, and secondary immune response may contribute to post-stroke cardiac dysfunction. | ^9^ |
| Male Wistar rats  (6 months) | Ischemia model via unilateral stereotaxic injection of endothelin-1 into the left/right Insular cortex | *4 weeks after stroke:*  Immunostaining performed for B-lymphocytes, T-lymphocytes, neutrophils, and leukocytes,  phosphorylated eNOS. | Neutrophil recruitment was significantly higher in stroke rats. T-lymphocyte infiltration was increased in the LA of stroke rats.  The level of LA leukocyte infiltration and the amount of LA fibrosis 28 days following stroke induction were significantly correlated. | Left and right insular ischemic strokes resulted in coronary microvascular endothelial dysfunction (CMED), myocardial inflammatory infiltration (MII), and fibrosis. MII was significantly correlated with CMED and fibrosis. | ^10^ |
| Male C57BL/6 J mice  (4–6 weeks) | Ischemia model via right MCAO | *4 weeks before and after MCAO:*  measure cardiac function;  *4 weeks after MCAO:*  gene expression of NLRP3, caspase-1, IL-1β;  NLRP3 and caspase-1 in myocardial tissue;  macrophage marker. | M1-polarized macrophage infiltration and NLRP3 inflammasome activation increased in the cardiac ventricle after diabetic stroke. The NLRP3 inflammasome inhibitor CY-09 restored cardiac function. | The M1-polarized macrophage-NLRP3 inflammasome activation is a pathway underlying the brain-heart interaction after diabetic stroke. | ^11^ |
| Male Wistar rats  (8–9 weeks) | Left/right model of cerebral embolization (polystyrene microspheres) | *7 days before surgery, 2 hours, 24 hours, and 7 days after surgery:*  measure cardiac function;  *2 hours and 24 hours after surgery:*  Measure plasma epinephrine, NE, and GDF-15;  *2 hours after surgery:*  the cardiac expression of eNOS, CAT, and GPX1 in mRNA and protein. | Following a stroke, circulating levels of catecholamines and GDF15 (growth differentiation factor 15) increased. Cerebral embolization altered nitro-oxidative stress signaling and impaired the myocardial expression of ADRB1 (adrenoceptor β1) and cardioprotective Survivor Activating Factor Enhancement signaling pathways. | The underlying molecular mechanisms of the stroke-induced myocardial alterations after cerebral embolization may involve the sympathetic nervous system or nitro-oxidative stress. | ^12^ |
| Rats | Ischemia model via right tMCAO | *3 months after MCAO:*  Cell death markers, including TNF alpha, Caspase 3, Fas Ligand, and MAP1LC3A. | Ischemic stroke animals displayed phenotypic expression of necrosis, apoptosis, and autophagy in their hearts. | Ischemic stroke was accompanied by cardiac myocyte death, indicating a close pathologic link between the brain and heart. | ^13^ |
| Rhesus  (8 years) | Ischemia model via transient global ischemia | *6 months after transient global ischemia:*  number of Purkinje cells,  apoptotic cells, astrogliosis, and TNF-α in the cerebellum,  expression of HLA-DR, caspase 3, TNF-α and CD68 in the heart,  expression of inflammatory markers in the granular cell layer of the cerebellum. | Significant Purkinje cell loss in lobule III and lobule IX of the TGI cerebellum relative to sham cerebellum, with corresponding upregulation of inflammatory and apoptotic cells. Similarly, TGI hearts revealed significant activation of inflammatory and apoptotic cells relative to sham hearts. | The current study supports a pathologic link between ischemia in the CNS and secondary cell injury in both distal brain regions (cerebellum) and peripheral organs (heart), which may further contribute to the delayed functional impairments in survivors of global ischemia. | ^14^ |

**References**

1 Min, J. *et al.* Cardiac dysfunction after left permanent cerebral focal ischemia: the brain and heart connection. *Stroke* **40**, 2560-2563, doi:10.1161/strokeaha.108.536086 (2009).

2 Veltkamp, R. *et al.* Experimental ischaemic stroke induces transient cardiac atrophy and dysfunction. *Journal of cachexia, sarcopenia and muscle* **10**, 54-62, doi:10.1002/jcsm.12335 (2019).

3 Bieber, M. *et al.* Stroke-induced chronic systolic dysfunction driven by sympathetic overactivity. *Annals of neurology* **82**, 729-743, doi:10.1002/ana.25073 (2017).

4 Chen, J. *et al.* MiR-126 Affects Brain-Heart Interaction after Cerebral Ischemic Stroke. *Translational stroke research* **8**, 374-385, doi:10.1007/s12975-017-0520-z (2017).

5 Butcher, K. S., Hachinski, V. C., Wilson, J. X., Guiraudon, C. & Cechetto, D. F. Cardiac and sympathetic effects of middle cerebral artery occlusion in the spontaneously hypertensive rat. *Brain research* **621**, 79-86, doi:10.1016/0006-8993(93)90300-c (1993).

6 Sun, L. *et al.* Aberration of L-type calcium channel in cardiac myocytes is one of the mechanisms of arrhythmia induced by cerebral ischemia. *Cellular physiology and biochemistry : international journal of experimental cellular physiology, biochemistry, and pharmacology* **22**, 147-156, doi:10.1159/000149792 (2008).

7 Wang, L. *et al.* Ionic mechanisms underlying action potential prolongation by focal cerebral ischemia in rat ventricular myocytes. *Cellular physiology and biochemistry : international journal of experimental cellular physiology, biochemistry, and pharmacology* **23**, 305-316, doi:10.1159/000218177 (2009).

8 Vornholz, L. *et al.* Acute Heart Failure After Reperfused Ischemic Stroke: Association With Systemic and Cardiac Inflammatory Responses. *Frontiers in physiology* **12**, 782760, doi:10.3389/fphys.2021.782760 (2021).

9 Yan, T. *et al.* Inflammatory responses mediate brain-heart interaction after ischemic stroke in adult mice. *Journal of cerebral blood flow and metabolism : official journal of the International Society of Cerebral Blood Flow and Metabolism* **40**, 1213-1229, doi:10.1177/0271678x18813317 (2020).

10 Balint, B., Jaremek, V., Thorburn, V., Whitehead, S. N. & Sposato, L. A. Left atrial microvascular endothelial dysfunction, myocardial inflammation and fibrosis after selective insular cortex ischemic stroke. *International journal of cardiology* **292**, 148-155, doi:10.1016/j.ijcard.2019.06.004 (2019).

11 Lin, H. B. *et al.* Macrophage-NLRP3 Inflammasome Activation Exacerbates Cardiac Dysfunction after Ischemic Stroke in a Mouse Model of Diabetes. *Neuroscience Bulletin* **36**, 1035-1045 (2020).

12 Meloux, A. *et al.* Ischemic Stroke Increases Heart Vulnerability to Ischemia-Reperfusion and Alters Myocardial Cardioprotective Pathways. *Stroke* **49**, 2752-2760, doi:10.1161/strokeaha.118.022207 (2018).

13 Ishikawa, H. *et al.* Ischemic stroke brain sends indirect cell death signals to the heart. *Stroke* **44**, 3175-3182, doi:10.1161/strokeaha.113.001714 (2013).

14 Acosta, S. A., Mashkouri, S., Nwokoye, D., Lee, J. Y. & Borlongan, C. V. Chronic inflammation and apoptosis propagate in ischemic cerebellum and heart of non-human primates. *Oncotarget* **8**, 102820-102834, doi:10.18632/oncotarget.18330 (2017).
